# Relationship Between Visual Perception and Participation Performance in Children with Autism Spectrum Disorder Aged 4–6 Years: A Cross-Sectional Study

**DOI:** 10.5041/RMMJ.10572

**Published:** 2026-04-26

**Authors:** Redkar Simran Sandeep, Ganapathy Sankar Umaiorubagam

**Affiliations:** 1 Ph.D. Scholar, SRM College of Occupational Therapy, SRM Institute of Science and Technology, SRM Nagar, Kattankulathur, Chengalpattu District, Tamil Nadu, India; 2 Dean, SRM College of Occupational Therapy, SRM Institute of Science and Technology, SRM Nagar, Kattankulathur, Chengalpattu District, Tamil Nadu, India

**Keywords:** Autism, human activities, occupational therapy, participation

## Abstract

**Objectives:**

This study explored the relationship between visual perceptual skills and participation performance in 4–6-year-old children diagnosed with autism spectrum disorder (ASD).

**Methods:**

A cross-sectional design was employed for children diagnosed with ASD. Visual perceptual abilities were assessed using the Motor-Free Visual Perception Test–Fourth Edition (MVPT-4), and participation levels were measured using the Children Participation Questionnaire (CPQ). Statistical analysis was done using SPSS version 26, employing one-sample *t*-tests and Pearson correlation analysis.

**Results:**

A total of 48 participants were included in the study: mean age, 4.98±0.82 years; 66.7% were male; 79.2% attended regular schools. One-sample *t*-tests indicated significant deficits across all CPQ dimensions (*P*<0.001). Visual perception was negatively correlated with autism severity (*r*=−0.429, *P*=0.002) and positively correlated with participation diversity (*r*=0.404, *P*=0.004). In the activities of daily living (ADL) and instrumental ADL (IADL) occupations, visual perception was significantly associated with all CPQ elements. Conversely, play and leisure occupations showed mostly negative correlations with the CPQ occupations, while social participation and education showed mixed results. Visual perception was positively correlated with most elements but negatively associated with enjoyment (*r*=−0.428; *P*=0.002).

**Conclusions:**

Preschool children with ASD demonstrate significant participation restrictions. Visual perception emerged as a critical determinant of participation, particularly in ADL and educational contexts. Early interventions targeting visual perception skills may enhance independence and functional engagement, though interventions should also address the enjoyment and emotional experience occupations to ensure holistic participation outcomes.

## INTRODUCTION

Participation, as defined by the World Health Organization, refers to an individual’s involvement in life situations and daily routines across different environments, including the home, community, and early educational settings.^1^ In early childhood, particularly between the ages of 4 to 6 years, participation is a dynamic and developmentally critical process through which children acquire foundational skills, gain independence, develop a sense of self, and build meaningful social relationships.^2^–^5^ Engaging in a wide range of age-appropriate activities not only enhances physical, cognitive, emotional, and social development but also supports school readiness and fosters self-esteem and emotional resilience.^6^ The preschool years represent a vital window of opportunity for early intervention, as children begin to assert autonomy, explore their environment with greater complexity, and prepare for the structured demands of formal education.

Children with autism spectrum disorder (ASD) often experience significantly reduced participation in daily activities compared to their typically developing peers.^7^–^10^ Autism spectrum disorder is a neurodevelopmental condition characterized by challenges in social communication, restricted and repetitive behaviors, and frequently co-occurring sensory processing difficulties, all of which can impact the child’s ability to engage meaningfully in various life situations.^11^ Research has consistently shown that children with ASD engage less frequently in play, social interaction, community activities, and daily living tasks, and the activities they do engage in may be limited in variety and often performed with lower levels of independence.^12^–^14^ These limitations can in turn have cascading effects on the child’s overall development, further reducing opportunities for skill acquisition, peer interaction, and emotional regulation.^15^–^17^

A growing body of literature has begun to explore the underlying factors contributing to reduced participation in children with ASD. Among these, visual perceptual skills have emerged as a potentially important but understudied underlying factor. Visual perception (VP) is defined as how the brain makes sense of visual information, relying on abilities like discrimination, spatial understanding, visual memory, and coordination of vision with movement.^18^ These abilities are critical for performing everyday tasks such as dressing, drawing, navigating spaces, recognizing objects, and engaging in structured and unstructured play.^19^,^20^ In preschool-aged children, whose learning is heavily dependent on visual exploration and imitation, deficits in VP may significantly restrict their ability to initiate, sustain, or enjoy participation in meaningful activities.

Children with ASD often present with VP deficits, which may manifest as difficulty understanding spatial relationships, poor coordination between vision and motor actions, or challenges in focusing on relevant visual stimuli while ignoring distractions.^21^–^23^ These VP challenges may interfere with their ability to engage in tasks that require matching, sorting, building, drawing, copying, or following visual instructions—activities that are commonly encountered in preschool and home environments. Furthermore, the interplay between VP and other core deficits of ASD, such as poor social attention or limited adaptive behavior, may further exacerbate participation difficulties. Despite its relevance, the relationship between VP and participation remains underexplored in younger children with ASD, particularly during the critical preschool years when foundational abilities are rapidly emerging.

Existing studies on participation in ASD have predominantly focused on older children and have typically examined only a few dimensions of participation, such as frequency or diversity. There is limited understanding of how preschool children with ASD participate across multiple dimensions such as independence, diversity, frequency, enjoyment, and parent satisfaction and how visual perceptual abilities may relate to these facets. Addressing this gap is essential, as early assessment and intervention targeting visual perceptual skills may serve to enhance participation and support more holistic developmental outcomes.

Therefore, the present study aimed to examine the participation patterns of preschool children with ASD aged 4–6 years and to explore the relationship between their VP skills and multiple dimensions of participation. By identifying how VP influences activity engagement, this study seeks to inform occupational therapy practices and early childhood interventions designed to support functional independence and quality of life for young children with ASD.

## METHODS

### Ethical Approval

The Institutional Ethical Committee of SRM Medical College Hospital & Research Centre, Kattankulathur, granted ethical approval (Approval No. 8494/IEC/2022).

### Study Design

This study employed a cross-sectional design to evaluate the relationship between visual perceptual abilities and occupational participation among children with ASD aged 4–6 years during the preschool period. A cross-sectional approach was carefully chosen to find the associations between visual perceptual abilities and participation outcomes during the preschool period. Convenience sampling was used to recruit ASD children from special schools and the SRM Autism Centre of Excellence, Kattankulathur, Tamil Nadu.

Children were diagnosed with ASD by a multidisciplinary team that included a developmental pediatrician, a clinical psychologist, and an occupational therapist. The Diagnostic and Statistical Manual of Mental Disorders, Fifth Edition^11^ and the Indian Scale for Assessment of Autism (ISAA)^24^,^25^ were used to confirm the diagnosis. Prior to confirming the diagnosis, routine medical screening, including vision and hearing assessments, was performed by a developmental pediatrician to exclude sensory impairment. Additionally, all participants were required to be receiving at least one therapy service, such as occupational therapy, speech therapy, or psychotherapy, or attending special education classes. Children were excluded if they presented with comorbid neurological conditions (e.g. epilepsy or cerebral palsy) or had uncorrected visual or hearing impairments.

Basic sociodemographic details (age, gender, school type, therapy services) were collected from parents. Potential confounding factors were considered in the analysis, including autism severity, sex, and school type. Written informed consent was obtained from parents prior to participation. Confidentiality of all data was ensured, and parents were informed of their right to withdraw from the study at any time without any consequences.

### Tools Used

#### Indian Scale for Assessment of Autism

The Indian Scale for Assessment of Autism (ISAA) is a culturally relevant tool developed to assess ASD in children and adolescents within the Indian context and widely used in clinical and research settings.^24^,^25^ The scale is recognized by the Government of India for disability certification and provides a comprehensive assessment of autism severity across multiple domains. Although internationally used tools such as the Childhood Autism Rating Scale are commonly applied, ISAA was selected in the present study due to its cultural relevance and widespread use in the Indian clinical context.

The ISAA scale consists of 40 items rated on a 5-point scale (1=never to 5=always) across six domains: social relationships and reciprocity, emotional responsiveness, language and communication, behavior patterns, sensory aspects, and cognitive components. Total scores range from 40 to 200, with 70–106 indicating mild autism, 107–153 moderate autism, and scores above 153 classified as severe autism. Cronbach’s alpha coefficients generally range from 0.85 to 0.95, indicating high internal consistency of the scale. The scale exhibits high stability over time, with test–retest reliability values typically above 0.80. Factor analyses support the theoretical structure of the scale, confirming its alignment with known autism constructs. Significant correlations with other established autism assessments (such as the Autism Diagnostic Observation Schedule [ADOS]) suggest good criterion validity. The ISAA has a sensitivity of 93.3% and specificity of 97.4%, but its level of agreement with the Childhood Autism Rating Scale is low (kappa coefficient of 0.14).

#### Children Participation Questionnaire

The Children Participation Questionnaire (CPQ) is a parent-reported tool designed to assess a child’s engagement in various activities.^26^ It measures participation across 44 activities within six occupations: activities of daily living, instrumental activities of daily living, play, leisure, social participation, and education. The CPQ is applicable to children aged 4–6 years (preschool) and 6–12 years (child). The questionnaire evaluates participation through five key measures: participation diversity, participation intensity, independence, child enjoyment, and parent satisfaction. The CPQ scores were the main outcome measure. The CPQ cut-off scores were expressed in terms of means (M). These thresholds were not publicly reported but were available upon request from the original test authors (private communication from Limor Rosenberg). The normative data for preschool children is expressed in terms of mean and standard deviation.^26^

The initial validation of CPQ yielded a Cronbach’s α coefficient of 0.79 (intensity), 0.89 (independence), 0.88 (child enjoyment), 0.90 (parent satisfaction).^26^ The test–retest reliability was estimated to be in the range of 0.84 to 0.90.^26^ The CPQ demonstrated temporal stability, with intra-class correlations ranging from 0.71 to 1.00. Significant differences were observed in all CPQ measures between children with disabilities and those without. The CPQ also effectively differentiated between different age groups and socio-economic statuses. Both convergent and divergent validity were confirmed.^26^

#### Motor-Free Visual Perception Test, Fourth Edition

The Motor-Free Visual Perception Test, Fourth Edition (MVPT-4) assesses visual–perceptual abilities across five items: spatial relationships, visual discrimination, figure–ground, visual closure, and visual memory, without requiring a motor response (referred to herein, as simply visual perception [VP]).^27^ It is standardized for individuals aged 4 to over 80 years. The test yields a total raw score, with no subscale scores available. A correct response is scored “1” while an incorrect response is scored “zero”. The total raw score is calculated on the total of correct responses. Psychometric properties indicate adequate reliability and validity, including internal consistency of 0.80, test–retest reliability of 0.76, and evidence of good content, criterion (*r*= 0.60), and construct validity.

### Data Analysis

Data analysis was performed using SPSS version 26.0, incorporating both descriptive and inferential statistical methods. Descriptive statistics were employed to summarize demographic variables. To assess the normality of the dataset, the Kolmogorov–Smirnov and Shapiro–Wilk tests were conducted. Since CPQ parameters and VP scores for children with ASD followed a normal distribution, one sample *t-*test was used for comparison. Pearson correlation analysis was conducted to assess the association between participation parameters and VP. A *P*-value of <0.05 was considered statistically significant.

## RESULTS

A total of forty-eight (*n*=48) children with ASD were recruited through convenience sampling from special schools and the SRM Autism Centre of Excellence, Kattankulathur, Tamil Nadu. The participants’ mean age was 4.98±0.82 years, with 66.7% being male. A majority (79.2%) of the children attended regular schools, while 20.8% were enrolled in special education settings.

**Figure 1:**
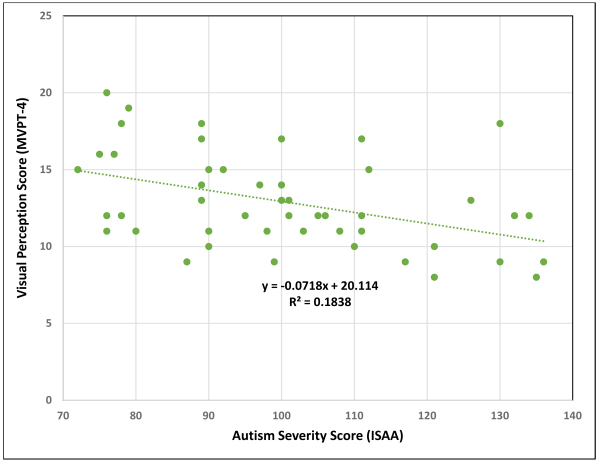
Correlation Between Visual Perception (VP) and Autism Severity Based on the Indian Scale for Assessment of Autism (ISAA)

**Figure 2:**
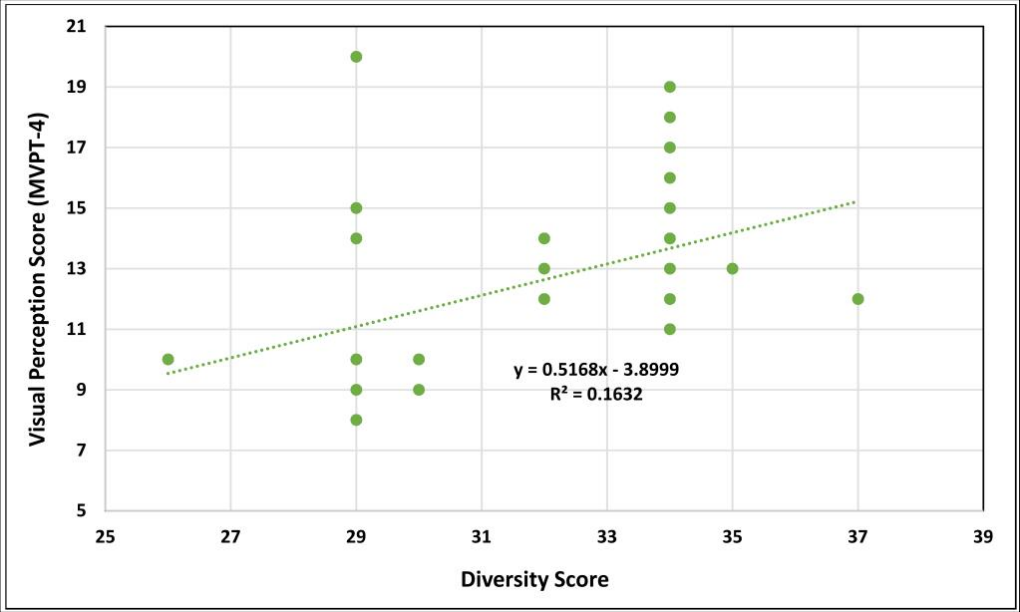
Correlation Between Visual Perception (VP) and the CPQ Diversity Score.

The average ISAA score among the participants was 100.45±18.22, indicating a predominance of mild to moderate autism severity. Table 1 provides an overview of the participants’ demographic details. Results from the one-sample *t*-test demonstrated statistically significant deficits across all parameters of the CPQ (*P*<0.01) (Table 2). Pearson correlation analysis revealed a significant negative relationship between VP and ISAA scores (*r*=-0.429, *P*=0.002) (Figure 1), and a significant positive correlation between VP and the diversity of participation (*r*= 0.404, *P*=0.004) (Figure 2) (Table 3).

**Table 1:** Demographic Distribution of Variables of the 48 Study Participants.

Variable	Values
Sex, *n* (%)	
Male	32 (66.7)
Female	16 (33.3)

School type, *n* (%)	
Regular	38 (79.2)
Special	10 (20.8)

Age, years, mean±SD	4.98±0.82
ISAA score, mean±SD	100.45±18.22
VP score, mean±SD	12.89±3.05

ISAA, Indian Scale for Assessment of Autism; VP, visual perception.

**Table 2:** One-sample *t*-test Analysis on CPQ Dimensions (*n*=48 participants).

CPQ Measures	Mean±SD	Cut-off Value of CPQ (Mean)	*t*-value	*P* value
Total CPQ				
Diversity	32.50±2.38	38	−15.957	<0.001
Intensity	1.78±0.18	3.86	−79.830	<0.001
Independence	1.33±0.34	5.05	−75.557	<0.001
Enjoyment	1.34±0.09	5.34	−281.028	<0.001
Parent satisfaction	1.04±0.09	5.22	−293.67	<0.001

ADL Domain				
Intensity	3.27±0.04	3.86	−92.620	<0.001
Independence	1.44±0.26	5.05	−95.418	<0.001
Enjoyment	1.65±0.08	5.34	−293.06	<0.001
Parent satisfaction	1.55±0.26	5.22	−97.103	<0.001

IADL Domain				
Intensity	1.31±0.52	3.86	−33.589	<0.001
Independence	0.83±0.21	5.05	−131.181	<0.001
Enjoyment	0.92±0.13	5.34	−233.864	<0.001
Parent satisfaction	0.72±0.13	5.22	−238.100	<0.001

Play Domain				
Intensity	1.46±0.23	3.86	−69.631	<0.001
Independence	1.53±0.28	5.05	−86.846	<0.001
Enjoyment	1.40±0.34	5.34	−78.168	<0.001
Parent satisfaction	1.68±0.34	5.22	−69.966	<0.001

Leisure Domain				
Intensity	1.13±0.12	3.86	−147.126	<0.001
Independence	1.25±0.17	5.05	−147.299	<0.001
Enjoyment	1.37±0.09	5.34	−278.217	<0.001
Parent satisfaction	1.00±0.19	5.22	−145.576	<0.001

Social Participation Domain				
Intensity	1.32±0.13	3.86	−128.203	<0.001
Independence	1.07±0.04	5.05	−582.929	<0.001
Enjoyment	1.30±0.17	5.34	−160.911	<0.001
Parent satisfaction	1.17±0.13	5.22	−214.695	<0.001

Education Domain				
Intensity	2.32±0.64	3.86	−16.422	<0.001
Independence	1.22±0.12	5.05	−211.827	<0.001
Enjoyment	1.33±0.05	5.34	−496.921	<0.001
Parent satisfaction	1.27±0.04	5.22	−643.147	<0.001

CPQ measures were evaluated against commonly accepted cut-off values^31^ (private communication from Limor Rosenberg).

ADL, activities of daily living; CPQ, Children Participation Questionnaire; IADL,

**Table 3:** Correlation Analysis Between Visual Perception, Age, Autism Severity, and CPQ Diversity Score.

Correlation Item		Age	Autism Severity	CPQ Diversity Score
**Visual Perception Score**	Pearson correlation	−0.058	−0.429	0.404
	Sig. (two-tailed)	0.697	0.002	0.004

CPQ, Children Participation Questionnaire.

Further occupation-specific correlations showed that VP was positively associated with all four CPQ elements within the activities of daily living (ADL) occupation—intensity (*r*=0.394, *P*=0.006), independence (*r*=0.407, *P*=0.004), enjoyment (*r*=0.407, *P*=0.004), and parent satisfaction (*r*=0.411, *P*= 0.004) measures (Table 4). Similarly, within the instrumental ADL (IADL) occupation, significant positive correlations were observed for intensity (*r*= 0.415, *P*=0.003), independence (*r*=0.417, *P*=0.003), enjoyment (*r*=0.411, *P*=0.004), and parent satisfaction (*r*=0.411, *P*=0.004). In contrast, the play occupation exhibited negative correlations across all elements—intensity (*r*=−0.300, *P*=0.038), independence (*r*=−0.376, *P*=0.009), enjoyment (*r*=−0.407, *P*=0.004), and parent satisfaction (*r*=−0.295, *P*= 0.042). Within the leisure occupation, negative correlations were found for independence (*r*=−0.306, *P*=0.035) and parent satisfaction (r=−0.364, *P*= 0.011), while intensity (*r*=−0.283, *P*=0.051) and enjoyment (*r*=0.018, *P*=0.904) showed non-significant associations. Social participation showed a mixed pattern, with positive correlations for intensity (*r*=0.369, *P*=0.010) and independence (*r*=0.352, *P*=0.014), but negative correlations for enjoyment (*r*=−0.412, *P*=0.004) and parent satisfaction (*r*=−0.417, *P*=0.003). In the education occupation, VP was positively correlated with intensity (*r*=0.404, *P*=0.004), independence (*r*=0.392, *P*=0.006), and parent satisfaction (*r*=0.342, *P*=0.017), while a significant negative correlation was observed for enjoyment (*r*=−0.428, *P*=0.002) (Table 4).

**Table 4:** Correlation Analysis Between Visual Perception (MVPT-4) and the CPQ Mean Measures Across the CPQ Occupations (*n*=48).

CPQ Occupations	Correlation	CPQ Measures			

		Intensity	Independence	Enjoyment	Parent Satisfaction
**ADL**	*r*	0.394	0.407	0.407	0.407
	*P* value	0.006	0.004	0.004	0.004

**IADL**	*r*	0.415	0.417	0.411	0.411
	*P* value	0.003	0.003	0.004	0.004

**Play**	*r*	−0.300	−0.376	−0.407	−0.295
	*P* value	0.038	0.009	0.004	0.042

**Leisure**	*r*	−0.283	−0.306	0.018	−0.364
	*P* value	0.051	0.035	0.904	0.011

**Social Participation**	*r*	0.369	0.352	−0.412	−0.417
	*P* value	0.010	0.014	0.004	0.003

**Education**	*r*	0.404	0.392	−0.428	0.342
	*P* value	0.004	0.006	0.002	0.017

All values present Pearson correlations (*r*).

ADL, activities of daily living; CPQ, Children Participation Questionnaire; IADL, instrumental

## DISCUSSION

The present study aimed to explore the relationship between VP and participation performance in children aged 4–6 years diagnosed with ASD. Among the 48 participants with ASD, 66.7% were male, which is consistent with global epidemiological trends suggesting a male-to-female ratio of approximately 4:1 in ASD diagnosis.^28^ In our study, 79.2% of the children were enrolled in regular schools, with only 20.8% attending special education settings. This may indicate a shift towards inclusive education models, where children with ASD are being integrated into mainstream environments. While inclusion can foster peer modeling and social exposure, it may also introduce challenges, particularly when environmental accommodations and teacher support are inadequate.^29^–^31^

### Correlations with Participation Diversity

Visual perception plays a fundamental role in early childhood functioning, as it underlies many task demands such as spatial orientation, object manipulation, and sequencing. The mean VP score in this study was significantly correlated with multiple aspects of participation. Visual perception was positively correlated with diversity of participation, suggesting that children with better visual perceptual skills engaged in a broader range of activities. This is supported by earlier findings linking visual-motor integration to task engagement and exploration in preschool children.^32^ A negative correlation was observed between VP and ISAA scores, indicating that children with higher autism symptom severity tend to have lower visual perceptual skills, which may in turn constrain participation. Similar associations between ASD severity and deficits in VP have been documented.^20^

### General Participation Performance

Results from the one-sample *t*-test revealed significantly lower mean scores for all participation dimensions (diversity, intensity, independence, enjoyment, and parent satisfaction) of the Children Participation Questionnaire (CPQ) (*P*<0.001) for all comparisons. These findings emphasize the widespread participation restrictions experienced by preschool children with ASD. Diversity of participation was significantly reduced, suggesting a limited range of engagement across daily life activities. Previous studies have similarly reported that individuals with ASD tend to engage in fewer types of activities compared to their typically developing peers, particularly in social, community, and recreational occupations.^33^

Intensity and independence scores were markedly lower across all occupations, highlighting the reduced frequency and self-sufficiency with which children with ASD engage in daily activities. These findings support existing evidence that children with ASD often require higher levels of adult support and engage less frequently in meaningful play or social routines.^9^ The extremely low enjoyment and parent satisfaction scores suggest that participation, even when it occurs, may not be fulfilling or enjoyable for either the child or caregiver. Emotional engagement is a critical dimension of participation, and its absence may further reduce motivation and reinforce withdrawal patterns.^34^

### Visual Perception and Participation Across Occupations

#### Activities of daily living and instrumental ADLs

In ADL and IADL, significant positive correlations were observed across all four parameters (intensity, independence, enjoyment, and parent satisfaction) with VP, reinforcing the importance of VP skills in executing and enjoying basic routines and tasks. These findings suggest that VP is a modifiable factor that can directly influence participation outcomes through occupational therapy. Although ADLs like dressing, grooming, and feeding are more structured and predictable than play or social activities, our data revealed low intensity, independence, and enjoyment, far below normative expectations in all parameters of participation.

Studies suggest that children with developmental disabilities reveal a positive correlation between VP and self-care tasks.^35^ Visual supports, routine training, and task-specific interventions are often necessary for success in these occupations. These findings are consistent with studies demonstrating poor adaptive functioning and dependence on caregivers with respect to participation in preschool children with ASD.^36^

The IADL occupation showed some of the lowest scores across all parameters, particularly independence and parent satisfaction. IADL tasks such as helping with chores, managing belongings, or interacting with the community require higher-order cognitive and motor integration, which are frequently impaired in ASD.^37^

#### Play and leisure

Play participation is a vital avenue for social, cognitive, and emotional development. In this study, children demonstrated very low enjoyment and independence in play, indicating significant impairments in initiating and sustaining imaginative or cooperative play. Prior literature confirms that play in children with ASD is often solitary, repetitive, and less symbolically rich. Similarly, leisure activities, which depend on intrinsic motivation and self-direction, showed the lowest scores in intensity and enjoyment, possibly due to sensory preferences, limited exploration, or environmental constraints.^38^ In play, VP was negatively associated with enjoyment and independence, which may appear counterintuitive. One interpretation is that children with higher VP abilities may also be more aware of their social or imaginative limitations in play contexts, thus reducing enjoyment. Alternatively, higher VP skills might lead to more rigid or rule-bound play patterns, which are less socially rewarding or interactive.^39^

#### Social participation and education

Social participation was severely restricted in our study, reinforcing the core challenges of ASD in social communication and reciprocal interaction. While structured settings like education showed slightly higher intensity scores, independence and enjoyment remained very low, reflecting the demands of early academic tasks and social expectations, which may be stressful for children with ASD.^40^ These findings underscore the need for social-emotional learning and inclusive pedagogies in early education settings.

In the leisure and social participation occupations, mixed correlations were found. For example, VP was negatively associated with enjoyment and parent satisfaction in the social and educational occupations. These findings point to a potential mismatch between performance competence and affective experience; children with adequate skills may still find participation stressful or unrewarding due to social anxiety, sensory overload, or lack of peer reciprocity.^21^

#### Clinical implications

This study underscored the multifaceted nature of participation in preschool children with ASD and highlights VP as a significant, though complex, contributor. Occupational therapy interventions should focus not only on enhancing visual perceptual abilities but also on promoting enjoyment and engagement through child-centered and play-based approaches, supporting families by enhancing parent satisfaction through collaborative goal-setting and contextual interventions, and integrating environmental adaptations in home and school settings to support autonomy and successful task completion.

### Study Limitations

This study had a small sample size (*n*=48), and participants were recruited through convenience sampling, which may limit generalizability. Confounding factors such as cognitive ability and socioeconomic background were not examined. In addition, reliance on parent-reported CPQ data may have introduced reporting bias. Future research is required, with larger, more diverse samples, longitudinal designs, and random or stratified sampling, to improve representativeness and generalizability.

## CONCLUSION

Preschool children with ASD show significantly reduced participation across all life occupations, with marked deficits in independence, enjoyment, and parent satisfaction. In our study, visual perception emerged as a critical factor influencing both the quantity and quality of participation, particularly in daily living and educational tasks. However, the nuanced findings such as inverse associations with enjoyment in social contexts suggest that interventions must be comprehensive, addressing not only skill-building but also affective experiences, environmental fit, and family expectations. These findings reinforce the importance of early, individualized, and holistic occupational therapy to optimize functional participation outcomes in young children with ASD.

## Abbreviations

ADL: activities of daily living
ASD: autism spectrum disorder
IADL: instrumental activities of daily living
CPQ: Children Participation Questionnaire
MVPT-4: Motor-Free Visual Perception Test–Fourth Edition
VP: visual perception
